# A modifiable universal cotinine-chimeric antigen system of NK cells with multiple targets

**DOI:** 10.3389/fimmu.2022.1089369

**Published:** 2023-01-13

**Authors:** Hee Young Kang, Soo Yun Lee, Hyun Min Kim, Su Ui Lee, Hyunseung Lee, Mi Young Cho, Se-Chan Oh, Seok-Min Kim, Hye Sun Park, Eun Hee Han, Seong-Eun Kim, Hyori Kim, Suk Ran Yoon, Junsang Doh, Junho Chung, Kwan Soo Hong, Inpyo Choi, Tae-Don Kim

**Affiliations:** ^1^Immunotherapy Research Center, Korea Research Institute of Bioscience and Biotechnology (KRIBB), Yuseong-gu, Daejeon, Republic of Korea; ^2^Research Center for Bioconvergence Analysis, Korea Basic Science Institute, Cheongju-si, Chungcheonbuk-do, Republic of Korea; ^3^Natural Medicine Research Center, KRIBB, Chungcheongbuk-do, Republic of Korea; ^4^Department of Functional Genomics, KRIBB School of Bioscience, Korea University of Science and Technology (UST), Daejeon, Republic of Korea; ^5^Department of Mechanical Engineering, Pohang University of Science and Technology (POSTECH), Gyeongbuk, Republic of Korea; ^6^Convergence Medicine Research Center, Asan Medical Center, Seoul, Republic of Korea; ^7^Department of Materials Science and Engineering, Research Institute of Advanced Materials (RIAM), Institute of Engineering Research, Bio-MAX institute, Seoul National University, Seoul, Republic of Korea; ^8^Department of Biochemistry and Molecular Biology, Cancer Research Institute, Seoul National University College of Medicine, Seoul, Republic of Korea; ^9^Graduate School of Analytical Science and Technology, Chungnam National University, Daejeon, Republic of Korea; ^10^Biomedical Mathematics Group, Institute for Basic Science (IBS), Daejeon, Republic of Korea; ^11^Department of Biopharmaceutical Convergence, School of Pharmacy, Sungkyunkwan University, Suwon, Republic of Korea

**Keywords:** cotinine, NK cells, CAR NK cells, universal, tumor

## Abstract

Natural killer (NK) cells are immune effector cells with outstanding features for adoptive immunotherapy. Immune effector cells with chimeric antigen receptors (CARs) are promising targeted therapeutic agents for various diseases. Because tumor cells exhibit heterogeneous antigen expression and lose cell surface antigen expression during malignant progression, many CARs fixed against only one antigen have limited efficacy and are associated with tumor relapse. To expand the utility of CAR-NK cells, we designed a split and universal cotinine-CAR (Cot-CAR) system, comprising a Cot-conjugator and NK92 cells (α-Cot-NK92 cells) engineered with a CAR containing an anti-Cot-specific single-chain variable fragment and intracellular signaling domain. The efficacy of the Cot-CAR system was assessed *in vitro* using a cytolysis assay against various tumor cells, and its single- or multiple- utility potential was demonstrated using an *in vivo* lung metastasis model by injecting A549-Red-Fluc cells. The α-Cot-NK92 cells could switch targets, logically respond to multiple antigens, and tune cytolytic activation through the alteration of conjugators without re-engineering. Therefore the universal Cot-CAR system is useful for enhancing specificity and diversity of antigens, combating relapse, and controlling cytolytic activity. In conclusion, this universal Cot-CAR system reveals that multiple availability and controllability can be generated with a single, integrated system.

## Introduction

Engineering chimeric antigen receptor (CAR)-natural killer (NK) and CAR-T cells against a tumor-associated antigens (TAA) is a promising strategy for the treatment of various tumors. The CAR has been designed to allow immune effector cells to recognize predominant antigens on tumor surfaces and kill their targets in a human leukocyte antigen (HLA)-unrestricted fashion (1). CARs usually consist of an extracellular antigen-binding domain (single-chain variable fragment (scFv) derived from a monoclonal antibody), a hinge region, a transmembrane domain, and an intracellular signaling domain (CD3ζ and costimulatory domains, including CD28, 4-1BB [CD137], or 2B4 [CD244]) (2).

Conventional CAR with a fixed antigen-specific scFv cannot target multiple TAAs. In some studies, the application of CAR therapy for solid demonstrated suboptimal outcomes because of tumor heterogeneity and the suppressive tumor microenvironment (3–6). Therefore, a second engineered CAR has been used to target the growth of resistant or relapsed tumors which lack the first targeted surface antigen or heterogeneous tumor cells with different TAAs (7).The conventional CARs have limitations in targeting diverse antigens and controlling CAR mediated-immune cell activation. To overcome these limitations, several universal and switchable CAR systems, including a flexible platform with multiple targeting mechanisms against heterogeneous TAAs and improved tunability, have been introduced with conjugators fused to biomolecules, including biotin (8, 9) fluorescein isothiocyanate (10) and peptide neo-epitope (11).

NK cells act as cytotoxic immune effectors that mediate short-lived rapid responses in rejecting tumors and virus-infected cells. Several clinical studies have reported that adoptively transferred allogeneic NK cells are safe for patients with hematologic and solid tumors (12, 13) are associated with the development of graft versus host disease (1) and require strict HLA matching. CAR-NK cells, as an alternative to autologous CAR-T cells, are used as off-the-shelf allogeneic therapeutics for immediate clinical use without associated cytokine release syndrome, as revealed in many CAR-T cell clinical trials (14–17).

Cotinine(Cot), a major metabolite of nicotine, is a small molecule with a molecular weight of 176.22 and is a hapten, which means it can only elicit an immune response when conjugated with a carrier protein. A daily dose of up to 1,800 mg of cotinine for four consecutive days demonstrated no deleterious side effects (18). The carboxyl group of carboxy-cotinine (trans-4-cotinincarboxylic acid) can be chemically crosslinked with an affibody or antibody (18), such as trastuzumab, cetuximab, and rituximab, which are clinically used to target TAAs. Our α-Cot scFv was obtained from a high-affinity antibody, that can bind to cotinine(concentration ranging from 1 ng/mL to 1 μg/mL) and has no cross-reactivity with structurally similar chemicals, including nicotine, anabasine, caffeine, and cholesterol (18).

To improve the antigen diversity, specificity, safety, and controllability of CAR-expressing NK cells, we developed a split and universal CAR system comprising NK92 (α-Cot-NK92) cells expressing a universal receptor (α-Cot-CAR) with scFv against cotinine (Cot) and a Cot-conjugator fused to a commercial affibody or antibody targeting TAA. Compared to the universal system wherein the tag is engineered into the switch or conjugator itself (19–21), our α-Cot-CAR systemsave the cost and time required for the conjugator manufacturing. The universal α-Cot-CAR can specifically respond to multiple antigens through various conjugators, and their activity can be regulated by the conjugator dose and α-Cot-NK92 cell lines with different CAR levels. The α-Cot-CAR system developed herein is conjugator-dependent and effective *in vivo*, suggesting the broad clinical potential of our system, which is different from that of previous studies (9, 21).

## Materials and methods

### Cell culture

Cancer cell lines were purchased from the American Type Culture Collection (ATCC, USA) and cultured inRPMI 1640 medium (Welgene, South Korea) supplemented with 10% heat-inactivated fetal bovine serum (FBS; Seradigm, USA) and antibiotic–antimycotic (Gibco, Waltham, MA, USA). The human NK cell line NK92 (CRL-2407; ATCC) was maintained in the ATCC-recommended medium. The proliferation rate was assessed using CellTrace™ Violet (ThermoFisher Scientific, Waltham, MA, USA) according to the manufacturer’s protocol. Cell viability was measured by trypan blue staining.

### CAR system

**Figure 1:**
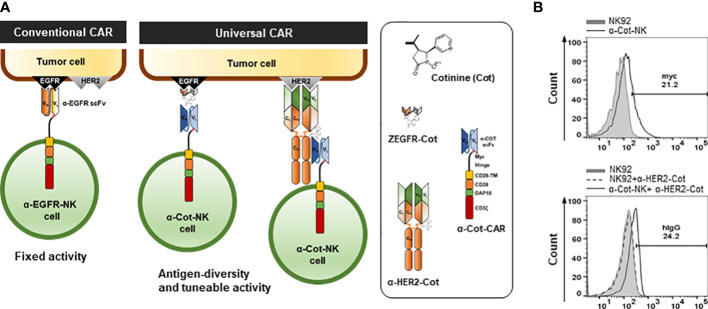
Engineering and establishment of α-Cot-NK92 cells as a universal CAR system **(A)** The conventional and universal CAR-NK system. **(B)** Levels of surface expression of CAR in NK92 or α-Cot-NK92 cells with/without α-HER2-Cot. The α-Cot-NK92 cells were cultured with puromycin for 3 d after α-Cot-CAR lentiviral transduction. Subsequently, transgene expression was evaluated using flow cytometry analysis (FACS) with α-Myc-PE (upper) and α-human IgG-PE conjugated antibody (hIgG, lower) after incubation with/without α-HER2-Cot. CAR, chimeric antigen receptor; Cot, cotinine; NK, natural killer.

A lentiviral vector(632155; Clontech) encoding α-Cot-Myc-CD8α-CD28TM-DAP10-CD3ζ was constructed (**Figure 1A**). The Cot-conjugators and scFv against Cot were obtained from a previous study (18).

### Flow cytometric analysis

Flow cytometric analysis was performed using a BD FACSCanto II cytometer(BD Biosciences, Franklin Lakes, NK, USA), and the data were analyzed using FlowJo software(Treestar Inc.Ashland, OR, USA). The cells were washed with cold FACS buffer and incubated with the following fluorochrome-conjugated mouse anti-human antibodies: NKp30 (CD337; 558408), NKp44 (CD336; 558563), NKp46 (CD335; 558051), NKG2D (CD314; 557940), granzyme B (561142), perforin (51-65995X), CD56 (555518); CD107a (555801), EGFR (563577), and ERK1/2 (pT202/pY204; 612592), purchased from BD Biosciences. The antibodies were diluted 1:100 and incubated for 30 min at 4°C in the dark. In addition, mouse IgG2b, κ isotype control (556437, BD Biosciences), Myc-Tag (9B11) (3739S, Cell Signaling Technology, Danvers, MA, USA), and HER-2 (2G11) (BMS120FI, eBioscience, Santa Clara, CA, USA) were used.

### Cytolysis assay

NK cell-mediated cytolysis was examined using calcein-AM (C1430, Thermo Fisher Science) as described previously (22) and the xCELLigence real time cell analysis system. Briefly, the target cancer cells were stained with calcein, and the stained cancer cells and NK cells were co-cultured according to the ratio of effector and target cells. The supernatant was taken 4 h later, the amount of calcein present in the supernatant was measured, and the xCELLigence real time cell analysis system was used to measure the extent to which attached target cells were suspended by NK cells by changing electrodes.

### Enzyme linked immunosorbent assay for measurement of IFN- γ or TNF-α

NK and cancer cells were mixed at a 1:1 ratio and incubated for 12 h. Cell-free supernatants were assayed for IFN-γ or TNF-α secretion using an ELISA kit (Cat No. 88-7316-88 and 88-7346-88, Invitrogen, Waltham, MA, USA) according to the manufacturer’s protocol.

### Cell-to-cell image analysis

The NK92 and AU565 cells were incubated with 1 µg/mL LysoSensor Green DND-153 (Invitrogen) for 30 min and 10 µg/mL CellTrace Far Red DDAO-SE (Invitrogen) for 15 min, respectively, at 37°C. The labeled NK92 cells in complete RPMI medium with 10 µg/mL propidium iodide (BD Biosciences) were seeded onto an anti-CD44 coated glass coverslip loaded in a Chamlide magnetic chamber (Live Cell Instrument, South Korea) (22).

### *In vivo* anti-tumor assays

All animal experiments were performed in accordance with guidelines of the Institutional Animal Care and Use Committee of the Korean Basic Science Institute (KBSI-AEC 1916). A549-Red-Fluc cells (Perkin-Elmer, Waltham, MA, USA) were intravenously injected into 6-week-old male *BALB/c-nu/nu* mice. The mice were injected with PBS, 10 mg/kg taxol, and α-Cot-NK92 cells through the tail vein and were subjected to weekly bioluminescence imaging (BLI; IVIS Spectrum, Perkin-Elmer, USA). For *ex vivo* analysis of BLI, the mice were sacrificed, and peripheral blood and lungs were harvested.

### Statistics

Data are presented as the mean ± SD or SEM of experimental replicates. One-way ANOVA was used to compare three or more independent groups using GraphPad Prism v5.03 software. *P*<0.05 was considered statistically significant.

## Results

### Engineering and establishment of α-Cot-NK92 cells as a universal CAR system

To allow the targeting of multiple TAAs through Cot-conjugators, α-Cot-CAR was engineered by fusing the extracellular domain (comprising scFv against Cot, Myc as an identifier of CAR expression, and a CD8-based hinge as a spacer in the formation of an immune synapse), a transmembrane domain originating from CD28, and intracellular signaling domains with cytoplasmic regions of CD28, DAP10, and CD3ζ (**Figure 1A**).

The intracellular signaling domains were determined by assessing the impact of each domain on CAR expression, cytolytic activity, and apoptotic cell death through the transient expression of CAR vectors comprising TM-10 (with only DAP10), TM-z (with only CD3z), and TM-10z (with both DAP10 and CD3z) together with a ZEGFR affibody instead of scFv (**Figure S1A**). The ΔEcto-TM-10z (without an affibody) was used as a negative control for the ectodomain. TM-z and TM-10z CARs containing CD3ζ exhibited higher cytolytic activity and CD107a (degranulation marker) expression than the others (**Figures S1C–E**). TM-z CAR exhibited the highest apoptotic cell death rate with the highest cytolytic activity. However, DAP10-containing TM-10 and TM-10z CARs exhibited reduced apoptotic cell death rates (**Figure S1F**). Therefore, CD3ζ and DAP10 play essential roles in cytolytic activity and cell survival.

To establish α-Cot-NK92 cells, α-Cot-CAR was transduced into NK92 cells using a lentiviral vector. Transduced NK92 cells were selected using puromycin for 3 days. The expression of α-Cot-CAR was confirmed by Myc in the extracellular domain of CAR using α-Myc-PE(**Figure 1B** upper) or after incubation of NK92 or transduced NK92 cells with α-HER2-cot, α-HER2 antibody bound to α-Cot-NK92 was identified using a human IgG-PE- conjugated antibody (**Figure 1B** lower). The α-Cot-NK92 cells expressed 20–25% more CAR in the extracellular region than the parental NK92 cells (**Figure 1B**). Further, the α-Cot-NK92 cells retained the characteristics of parental NK92 cells including NK-specific activating receptor profiles, such as NKp30, NKp44, NKp46, and NKG2D (**Figure S2A**); functional markers, such as IFN-γ, granzyme B, and perforin (**Figure S2B**); proliferation rate (**Figure S2C**); and viability (**Figure S2D**).

### The action of α-Cot-NK92 cells depends on the type of conjugator

**Figure 2:**
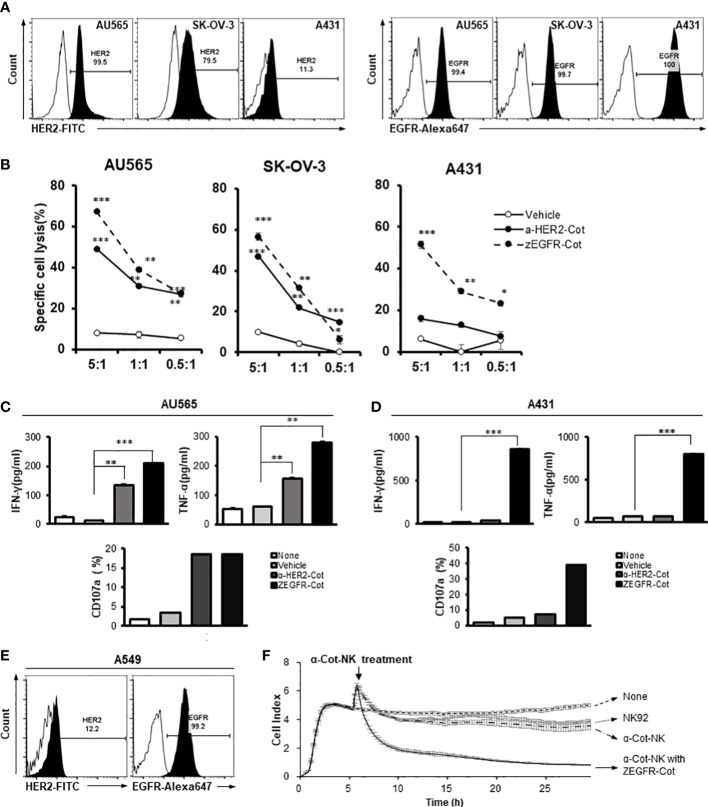
The action of α-Cot-NK92 cells depends on the type of conjugator. **(A)** Expression levels of HER2 and EGFR in various tumor cell lines, including mammary gland adenocarcinoma (AU565), ovarian adenocarcinoma (SK-OV-3), and epidermoid carcinoma (A431), were confirmed through FACS. Histograms are representative of at least three independent experiments. **(B)** Cytotoxicity against HER2^+^, EGFR^+^, or HER2^+^ and EGFR^+^ tumor cells mediated by α-Cot-NK92 cells with or without α-HER2-Cot or ZEGFR-Cot. The α-Cot-NK92 cells were co-cultured with calcein-stained tumor cells with or without a conjugator for 4 h at E:T ratios of 5:1, 1:1, and 0.5:1. The intensity of fluorescence emitted from the lysed target cells was measured using a fluorescence plate reader (SpectraMax i3x). All cytotoxicity data are represented as the mean ± S.D. of triplicate experiments. The statistical significance of differences between groups was evaluated using paired Student’s *t-*test. ***P < 0.001; **P < 0.01; * *P* < 0.05 vs. vehicle; ns: not significant. **(C, D)** IFN-γ and TNF-α secretion and CD107a expression in α-Cot-NK92 cells cultured with or without HER2^+^EGFR^+^ AU565 **(C)** or HER2^−^EGFR^+^ A431 **(D)** cells and with/without the conjugator (α-HER2-Cot or ZEGFR-Cot). Secreted IFN-γ and TNF-α levels were measured *via* ELISA using the medium of α-Cot-NK92 cells co-cultured with target cells at a 1:1 E:T ratio for 12 h. All experiments were performed in triplicate wells for each condition and repeated at least two times. Average values are shown as the mean ± S.D. of triplicates. Statistical significance of differences between groups was evaluated using paired Student’s *t*-test. ***P < 0.001; **P < 0.01; ns: not significant. CD107a expression in α-Cot-NK92 cells was evaluated through FACS analysis after co-culture with target cells at a 5:1 E:T ratio for 4 h. Each value represents the percentage of CD56^+^CD107a^+^ cells in flow cytometric density plots. **(E)** Expression level of HER2 and EGFR in pulmonary adenocarcinoma (A549) confirmed through FACS analysis. Histograms are representative of at least three independent experiments. **(F)** Kinetics of α-Cot-NK92 cell-mediated tumor cell lysis using the xCELLigence real time cell analysis system. NK92 or α-Cot-NK92 cells were co-cultured with unlabeled A549 cells with/without ZEGFR-Cot at a 5:1 E:T ratio and monitored over time. CAR, chimeric antigen receptor; Cot, cotinine; NK, natural killer.

To assess the antigen-specificity of α-Cot-CAR, α-Cot-NK92 cells were co-cultured *in vitro* with three tumor cell lines: AU565 (mammary gland adenocarcinoma), SK-OV-3 (ovary adenocarcinoma), and A431 (skin epidermal carcinoma). The surface expression of HER2 and EGFR was evaluated by flow cytometry. The adenocarcinoma cell lines AU565 and SK-OV-3 expressed high levels of HER2 and EGFR, whereas the carcinoma cell line A431 expressed low levels of HER2 and high levels of EGFR (**Figure 2A**).

Using an affibody(ZEGFR)-based conjugator against EGFR(ZEGFR-Cot) or antibody(trastuzumab)-based conjugator against HER2(α-HER2-Cot), we evaluated the cytolytic activity of α-Cot-NK92 cells. We found that the α-Cot-NK92 cells exhibited cytolytic activity according to the antigen-specificity of the conjugator, regardless of tissue specificity, in various tissue-derived tumor cells. Upon exposure of tumor cells to α-Cot-NK92 cells with α-HER2-Cot, the cytolytic activity of α-Cot-NK92 cells was positively correlated with HER2 expression levels in the tested cell lines. Due to the high levels of EGFR in the tested cell lines, α-Cot-NK92 cells with ZEGFR-Cot exhibited high cytotoxicity (45%–70%; **Figure 2B**). The α-Cot-NK92 cells, were co-cultured with AU565 or A431 cells with or without a conjugator to evaluate their cytokine secretion and degranulation functions. The secretion of IFN-γ and TNF-α and the expression of the degranulation marker CD107a in α-Cot-NK92 cells increased when α-HER2/ZEGFR-Cot was added in co-culture with AU565 cells expressing both HER2 and EGFR (**Figure 2C**), and when ZEGFR-Cot was added to co-culture with A431 cells expressing low levels of HER2 and high levels of EGFR (**Figure 2D**). The increase in activity following the addition of the conjugator was mediated by ERK phosphorylation (**Figure S3**).

We investigated the cytolytic kinetics of α-Cot-NK92 cells with and without a conjugator using the xCELLigence real-time cell analysis system to measure the impedance of the attached target cell. A dimensionless cell index indicated the number of surviving lung carcinoma A549 cells with a small amount of HER2 and high EGFR expression on the cell surface (**Figures 2E, F**). A rapid decrease in cell index <4 h after the addition of α-Cot-NK92 cells with ZEGFR-Cot indicated successful A549 cell lysis, whereas no significant cytolytic effect was detected in parental NK92 and α-Cot-NK92 cells in the absence of conjugators (**Figure 2F**). This suggests that α-Cot-NK92 cells can react with diverse conjugators and exhibit ligand-dependent cytotoxicity, mediated by the conjugator, against tumor cells.

### Combination with conjugator enhances recognition of multiple antigens by α-Cot-NK92 cells

**Figure 3:**
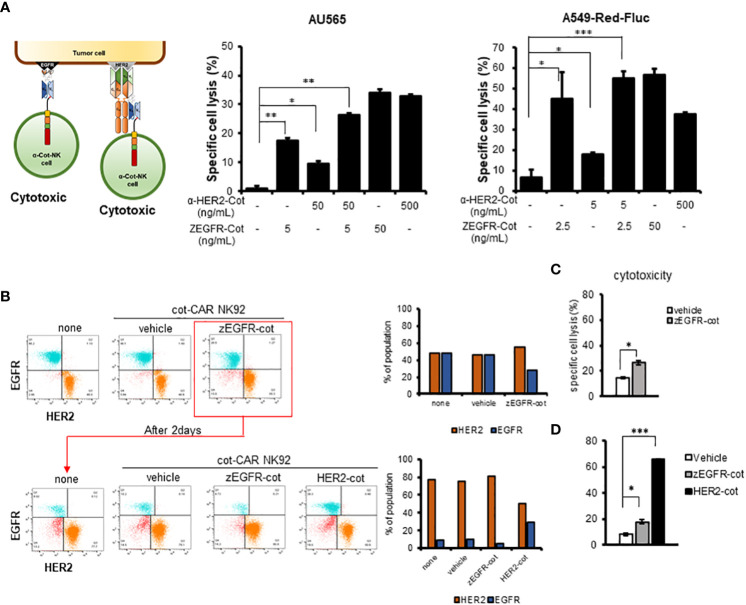
Combination with conjugator enhances recognition of multiple antigens by α-Cot-NK92 cells. **(A)** Additive cytotoxic effects of α-Cot-NK92 cells in multi-targeting through co-treatment with α-HER2-Cot and ZEGFR-Cot. α-Cot-NK92 cells were co-cultured with calcein-stained AU565 cells with/without α-HER2-Cot (50 or 500 μg/mL), ZEGFR-Cot (5 or 50 ng/mL), or both α-HER2-Cot and ZEGFR-Cot at a 1:1 E:T ratio for 4 h, or were co-cultured with calcein-stained A549-Red-Fluc cells with/without α-HER2-Cot (5 or 500 μg/mL) and/or ZEGFR-Cot (2.5 or 50 ng/mL) at a 5:1 E:T ratio for 4 h. **(B)** To mimic the heterogeneity of the recurrent tumor, the EGFR^+^HER2^−^ MDA-MB-231 and EGFR^−^HER2^+^ MDA-MB-453 cells were mixed. On day 1, one group was not treated with α-Cot-NK92 cells (none) and the other groups were co-cultured with α-Cot-NK92 cells with/without ZEGFR-Cot at a 1:1 E:T ratio for 4 h. After removing α-Cot-NK92 cells, the remaining target cells were cultured for 2 d and then co-cultured with the α-Cot-NK92 cells with ZEGFR-cot or α-HER2-cot. **(C)** Cytotoxicity of α-Cot-NK92 cells to tumor cells on day 1 and **(D)** after 2 d in a model mimicking a recurrent tumor. Population change was measured using a FACS. All cytotoxicity data are presented as the mean ± S.D. of triplicates. Statistical significance of differences between groups was evaluated using paired Student’s *t-*test. ***P < 0.001; **P < 0.01; *P < 0.05. CAR, chimeric antigen receptor; Cot, cotinine; NK, natural killer. α-Cot-NK92α-Cot-NK92α-Cot-NK92α-Cot-NK92α-Cot-NK92α-Cot-NK92α-Cot-NK92.

α-Cot-CAR was designed as a tunable receptor with the ability target multiple antigens. Using ZEGFR-Cot and α-HER2-Cot, we evaluated the cytolytic activity of α-Cot-NK92 cells in a dose-dependently manner. The cytolytic activity of α-Cot-NK92 cells increased with the conjugator dose and reached a maximum at approximately 1 μg/mL α-HER2-Cot or 0.05 μg/mL ZEGFR-Cot. (**Figure S4**). To identify the ability of α-Cot-NK92 cells to target dual antigens in AU565 cells, 50 ng/mL α-HER2-Cot and 5 ng/mL ZEGFR-Cot (which represent 9.5% and 17.6% of maximal cytolytic effects, respectively) were administered individually or in combination. Combination treatment showed an additive effect, with approximately 26.4% of the maximal cytolytic effect in AU565 cells (**Figure 3A**). The dual-targeting potential of α-Cot-NK92 cells was evaluated in A549-Red-Fluc cells expressing low HER2 and high EGFR levels (**Figure S5A**). Upon treatment with 5 ng/mL α-HER2-Cot or 2.5 ng/mL ZEGFR-Cot, α-Cot-NK92 cells exhibited 17.7% or 44.8% of the maximal cytolytic effect of each conjugator, respectively. Combination treatment with both conjugators resulted in a cytolytic effect comparable to that of 50 ng/mL ZEGFR-Cot and an enhanced cytolytic effect compared with that of 500 ng/mL α-HER2-Cot alone (**Figure 3A**). This suggests that α-Cot-NK92 cells can kill tumor cells expressing multiple antigens at different levels through dual or multiple targeting of various antigens using a small amount of conjugators.

Due to the heterogeneity of tumors, recurring tumors exhibit different characteristics compared to the original tumors. For example, exposure of HER2+HER3+ MDA-MB-453 cells to α-Cot-NK92 cells with α-HER2-Cot for 4h, followed by culture for 96h, decreased the HER2+HER3+ population by <10%, and increase the HER2-HER3+population by >90%. (**Figures S6**, **3B**).

Based on these results, a recurrent tumor model system mimicking the heterogeneity of recurrent tumors was constructed by mixing EGFR^+^HER2^−^ MDA-MB-231 and EGFR^-^HER2^+^ MDA-MB-453 cells. To evaluate the cytotoxicity of α-Cot-NK92 cells, we determined the percentage of reduced EGFR+ or HER2+ values in the group with or without the conjugator(s) compared to the untreated group (none) (**Figure 3B**). Following treatment of α-Cot-NK92 cells with ZEGFR-Cot on mixed target cells for 4 h, cytotoxicity was increased compared to the group without the conjugate (**Figure 3C**), and the population ratio of the remaining EGFR+ target cells was decreased compared to the untreated group After removing the α-Cot-NK92 cells, the remaining target cells were cultured for 2 days, and the α-Cot-NK92 cells were treated with ZEGFR-cot ​​or HER2-cot, respectively, to determine the cell population ratio and cytotoxicity. As a result, compared with the untreated group, the population ratio of HER2+ cells was decreased (**Figure 3B**) and the cytotoxicity was increased in the α-HER2-Cot treated group, whereas that of the ZEGFR-Cot treated group did not change (**Figure 3D**). This implies that dual or multiple targetable α-Cot-NK92 cells can be effectively applied to heterogeneous recurrent tumor cells without re-engineering CARs.

### α-Cot-NK92 cells act in a conjugator-dependent manner at the immune synapse

**Figure 4:**
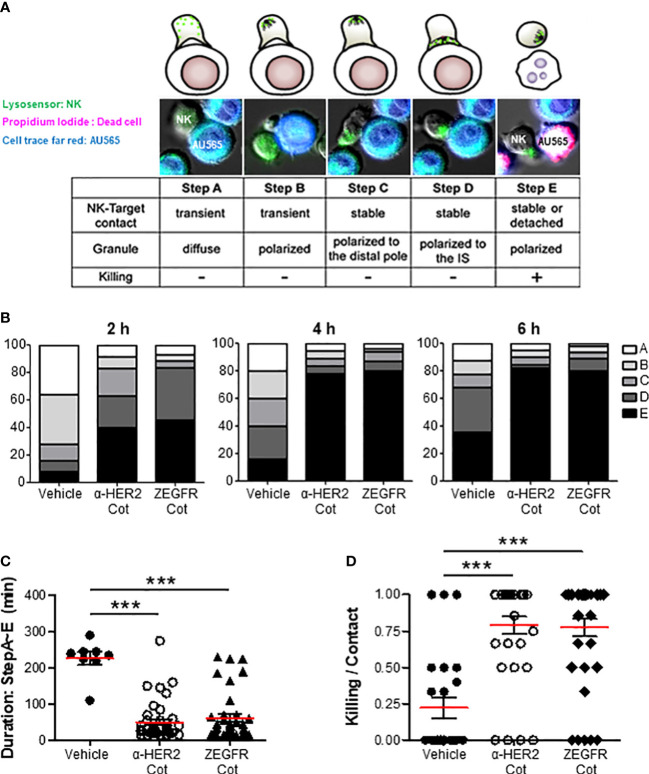
α-Cot-NK92 cells acts in a conjugator-dependent manner at the immune synapse **(A)** Images depicting the sequential steps in the interaction between α-Cot-NK92 and AU565 cells with or without conjugators (α-HER2-Cot and ZEGFR-Cot). The α-Cot-NK92 and AU565 (HER2^+^EGFR^+^) cells were labeled with LysoSensor Green and DDAO-SE, respectively, and co-cultured in a medium containing propidium iodide to assess the lytic dynamics of α-Cot-NK92 cells with or without conjugators. Images were acquired using a fluorescence microscope. The snapshots are provided in Supplementary Mov1. **(B)** Percentage of α-Cot-NK92 cells remaining at each step after 2, 4, or 6 h of co-incubation with AU565 cells with/without conjugators. **(C)** Duration of the process, and **(D)** ratio of α-Cot-NK92 cells that kill target cells for target-contacting cells. Data are shown as the mean (red line) ± S.D. Statistical significance of differences between groups was evaluated using paired Student’s *t-*test. ***P < 0.001. Cot, cotinine; NK, natural killer.

The immunological synapse between an NK cell and its target cell is the dynamic interface that allows for the sequential stages, including the initiation, effector, and termination stages of NK cells resulting in the lysis of the target cells. In the initial stage, the NK cells contact the target cells using diffused (**Figure 4A**, step A) or polarized granules (**Figure 4A**, step B). In the effector stage, NK cells form a stable cleft wherein cytolytic molecules are secreted, recruit lytic granules to the distal poles (**Figure 4A**, step C), and transport them into the immunological synapse (**Figure 4A**, step D). In the termination stage, the NK cells kill target cells with membrane blebs, increase propidium iodide fluorescence, and detach from the target cells (**Figure 4A**, step E). The percentage of α-Cot-NK92 cells remaining at each step was measured at 2, 4, and 6 h after co-culture with AU565 cells containing α-HER2-Cot or ZEGFR-Cot, with or without a conjugator (**Figure 4B**). Following the addition of a conjugator. α-Cot-NK92 cells rapidly entered the effector and termination stage 2 h after co-culture with AU565 cells, approximately 80% of α-Cot-NK92 cells were in the termination stage at 4, and 6 h after co-culture, the duration from steps A to E was much shorter (**Figure 4C**), and the killing ratio of α-Cot-NK92 cells on contacted target cells was approximately 75% (**Figure 4D**). These results indicate that α-Cot-NK92 cells act in a conjugator-dependent manner on tumor cells and rapidly induce cytolysis (Mov.S1).

### α-Cot-NK92 cells with a conjugator inhibit tumor growth in *an in vivo* lung cancer model

**Figure 5:**
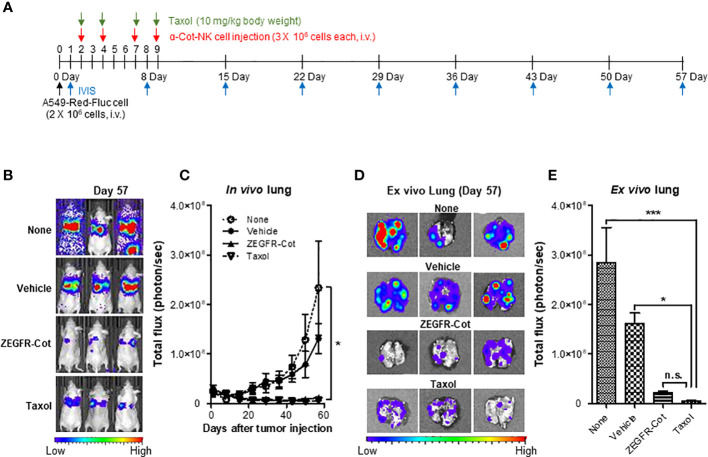
α-Cot-NK92 cells with a conjugator inhibit tumor growth in *an in vivo* lung cancer model. **(A)** The schedule of *in vivo* studies using luciferase-expressing A549-Red-Fluc cells in mouse metastasis model treated with α-Cot-NK92 cells with or without ZEGFR-Cot. **(B)** Representative *in vivo* bioluminescence imaging of each group treated with α-Cot-NK92 cells with or without ZEGFR-Cot and Taxol (10 mg/kg body weight) compared to levels in the untreated group (none) on day 57 after tumor cell injection. **(C)** The tumor burden of each group (n = 10) in *in vivo* lungs quantified as total flux (photon/s), which was monitored weekly for 57 d after the A549-Red-Fluc injection. **(D, E)** Representative BLI **(D)** and total quantitative flux **(E)** in *ex vivo* lungs of each group extracted on day 57 after *in vivo* BLI. ***P < 0.001 vs. none; *P < 0.05 vs. vehicle; Tukey’s multiple comparison test; n.s.: not significant. Bioluminescent images were acquired after an i.p. injection of D-luciferin (150 mg/kg body weight) and analyzed using the Living Image Software. Total flux data were plotted as mean ± SD. Cot, cotinine; NK, natural killer.

We induced the formation of lung metastases, in which tumor cells are trapped within the lung microvasculature, by injecting 2 × 10^6^ A549-Red-Fluc cells into the tail vein of BALB/c nude mice. On days 2, 4, 7, and 9 after cancer cell inoculation, nude mice bearing A549-Red-Luc cells were i.v. injected with 3 × 10^6^ α-Cot-NK92 cells, which were pre-incubated with/without ZEGFR-Cot (0.1 μg/mL) for 1 h, and metastatic lung tumor development was monitored weekly from day 1 after tumor inoculation using *in vivo* BLI (**Figure 5A**). PBS and Taxol was administered to mice as negative and positive controls, respectively, to achieve anti-tumor effects. Although α-Cot-NK92 cells without ZEGFR-Cot had little effect on metastatic tumor development compared to the untreated group, the administration of α-Cot-NK92 cells with ZEGFR-Cot or Taxol was effective and significantly reduced the remaining tumor restricted to small areas with low intensity in the lung (**Figures 5B, C**). For BLI of excised lungs, α-Cot-NK92 cells with ZEGFR-Cot or Taxol had significant regressive effects on the formation and area of metastatic tumor nodules compared to the untreated or α-Cot-NK92 cells without conjugator groups (**Figures 5D, E**). The results showed that α-Cot-NK92 cells with a conjugator can significantly inhibit lung tumor burden *in vivo*.

We then investigated whether α-Cot-NK92 cells with a conjugator could migrate toward the tumor site *in vivo*. A human skin cancer xenograft model was established by subcutaneous (s.c.) inoculation of A431 (skin epidermal carcinoma) cells into the right flanks of nude mice. On day 7 after tumor inoculation, NK92 or α-Cot-NK92 cells with or without ZEGFR-Cot cells were labeled with 1,1’-dioctadecyl-3,3,3’,3’-tetramethylindotricarbocyanine iodide (DiR), and the DiR-labeled NK cells were intravenously (i.v.) injected through the tail vein. Mice were euthanized 72 h after NK cell injection to collect subcutaneous tumors, and DiR fluorescence signal intensity was quantified in the tumors after treatment with α-Cot-NK92 cells with ZEGFR-Cot and α-Cot-NK92 cells injected with ZEGFR-Cot separately was 1.5- and 1.2-fold higher than that with NK92 cells, respectively (**Figure S7**). This indicates that α-Cot-NK92 cells with ZEGFR-Cot could migrate toward the tumor site and remain there.

### Monoclonal α-Cot-NK92 cell lines have different killing potential

**Figure 6:**
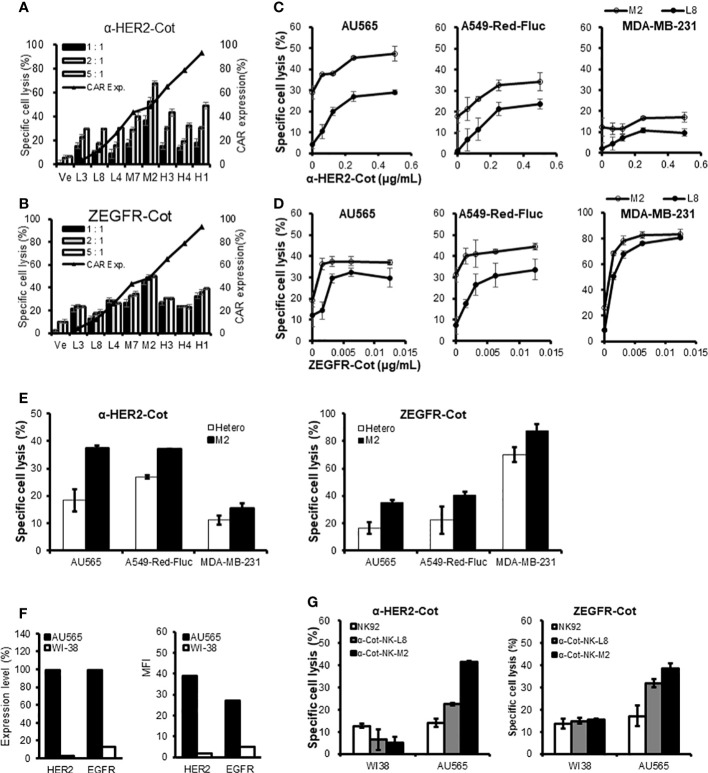
Monoclonal α-Cot-NK92 cell lines have different killing potentials. **(A, B)** α-Cot-NK92 clones were established through single cell sorting using FACS in an expression level-dependent manner for CAR-Myc. Black triangles with a line indicate the expression level of CAR-Myc. The ability of α-Cot-NK92 clones to lyse AU565 cells was assessed using co-culture with α-HER2-Cot (A: 500 ng/mL) or ZEGFR-Cot (B: 50 ng/mL) for 4 h at E:T ratios of 5:1, 2:1, and 1:1. Ve: vehicle. **(C, D)** Comparison of cytolytic potency between low (L8) and medium (M2) CAR-expressing α-Cot-NK92 clones through co-culture with AU565, A549-Red-Fluc, or MDA-MB-231 cells in a dose-dependent manner of α-HER2-Cot (C: from a concentration of 500 to 62.5 ng/mL, upper panel) or ZEGFR-Cot (D: from a concentration of 12.5 to 1.56 ng/mL, lower panel). **(E)** Comparison of cytolytic potency between M2 clone and heterogeneous α-Cot-NK92 cells through co-culture with AU565, A549-Red-Fluc, or MDA-MB-231 cells with α-HER2-Cot (125 ng/mL) or ZEGFR-Cot (6.25 ng/mL). **(F)** Relative expression level (%) and mean fluorescence intensity (MFI) of HER2 and EGFR in breast cancer cells (AU565) and normal lung cells (WI-38). **(G)** Cytolytic activity of NK92, monoclonal α-Cot-NK92-L8, and α-Cot-NK92-M2 cells with ZEGFR-Cot or HER2-Cot against AU565 and WI-38 cells. CAR, chimeric antigen receptor; Cot, cotinine; NK, natural killer.

The conjugator`s type, dose, and composition are the key factors that control the target reactivity and cytolytic activity of universal α-Cot-NK92 cells. α-Cot-NK92 cells transfected *via* lentiviral infection are heterogeneous and contain various levels of CAR and surface receptor repertoires. To modulate the killing potential of α-Cot-NK92 cells, monoclonal α-Cot-NK92 cell lines were classified according to CAR expression level through single-cell sorting using flow cytometry. The cytolytic activity of established monoclonal α-Cot-NK92 cell lines with HER2-Cot or ZEGFR-Cot was evaluated and showed a tendency to increase with CAR-Myc expression (black triangles with a line; **Figures 6A, B**). However, some monoclonal α-Cot-NK92 cell lines with high CAR levels showed lower cytolytic activity than M2 macrophages with moderate CAR levels (**Figures 6A, B**).

Monoclonal α-Cot-NK92-M2 cells showed higher basal cytolytic activity and potential than monoclonal α-Cot-NK92-L8 cells co-cultured with AU565, A549-Red-Fluc, or MDA-MB-231 cells in a dose-dependent fashion (**Figures 6C, D**). The MDA-MB-231 cells showed lower HER2 expression than of A549-Red-Fluc cells did (**Figure S5**). In addition, α-Cot-NK92-M2 cells showed a higher basal cytolytic activity than heterogeneous α-Cot-NK92 cells (**Figure 6E**). We evaluated the cytolytic activity of the monoclonal cell lines against normal lung (WI38) and breast cancer (AU565) cells. The WI-38 cells expressed 3.08% (Mean Fluorescent Intensity (MFI) 1.82) and 13.1% (MFI 4.79) HER2 and EGFR, respectively, relative to the IgG-stained cells. The AU565 cells expressed 99.5% (MFI 38.99) HER2 and 98.9% (MFI 26.90) EGFR relative to IgG-stained cells (**Figure 6F**). The α-Cot-NK92-L8 and α-Cot-NK92-M2 cell lines exhibited specific cytotoxicity against AU565 cells, and lower or similar cytolytic activity to parental NK92 cells compared with WI38 cells (**Figure 6G**), indicating that monoclonal α-Cot-NK92-L8 and α-Cot-NK92-M2 cell lines have tumor-specific killing potential applicable to tumor traits.

## Discussion

CAR therapies using T and NK cells target and destroy tumor cells by recognizing TAAs which are expressed on the tumor surface. Despite the tremendous success of CAR-T cells against CD19-expressing B cell leukemia (23, 24), conventional CAR methods have several limitations for clinical applications. Due to the diverse TAA and heterogeneity of tumor cells, conventional CARs, which cannot target more than one antigen on tumor cells, may promote more preferential and aggressive growth of targeted antigen-negative tumor cells. In addition, the identification and engineering of scFv against various TAAs are time-, cost-, and labor-intensive (10).

Herein, we developed a novel universal CAR-NK system wherein gene-engineered NK cells acquire specificity to cotinine-fused antibodies or antibody mimetics (conjugators) and eliminate tumors by conjugator-dependent recognition of various TAAs. To enhance the specificity, flexibility, and controllability of CARs, the universal CAR-NK system is comprised of α-Cot-NK92 cells and conjugators with Cot. The functional activity of α-Cot-NK92 cells was evaluated using a conjugator against EGFR and HER2, which are well-studied TAAs. Overexpression of EGFR and HER2 occurs in various human tumors, including prostate, brain, lung, and breast tumors, and is implicated in tumor development. EGFR has been found in 40%–80% of patients with non-small cell lung cancer and both premalignant and malignant lung tumors (25). HER2 is less common than EGFR, is a significant predictor of decreased overall survival in patients with breast cancer, and is associated with poor prognosis in patients with non-small cell lung cancer (26).

The α-Cot-NK92 cells with ZEGFR/α-HER2-Cot recognized and lysed tumor cells depending on EGFR/HER2 surface expression levels in various tissue-derived cancer cell lines including AU565, SK-OV-3, A431, and A549 cells. This indicates that the α-Cot-NK92 system works in an antigen-specific manner with a conjugator against a specific TAA, the cytolytic activity is not limited to the cancer of specific tissues and can act against various cancer cells. Additionally, we employed our α-Cot-CAR system to target one more antigen by dual treatment with a small amount of conjugator without genetic manipulation of NK cells and lysed cancer cells in an additive or synergistic manner using *in vitro* assays and *in vivo* lung metastasis models. Our finding suggest that α-Cot-NK92 cells can be compatible and promote antigen diversity with various types of conjugators and can save the cost and time required for re-engineering CARs. Moreover, given the diversity in the composition of TAA in tumors, this method could be applied clinically for patient-specific therapeutics.

Due to the compromised anti-tumor immunity in patients with tumors as a results of immunosuppressive or escape mechanisms facilitated by the tumor microenvironment, adoptive cancer immunotherapy with NK cells is preferred. Donor-derived allogeneic NK cells do not recognize tumor cells as “self,” unlike immune cells (27, 28). Because allogeneic NK cells have a shorter *in vivo* half-life than T cells in clinical trials, NK cells are considered suitable for genetic modification, as with CAR constructs and other immunomodulatory functions. NK cells are more effective when TAA targets are present at low levels in normal tissues (29, 30). Based on our results, α-Cot-NK92 cells treated with ZEGFR/HER2-Cot showed specific cytolytic activity against AU565 cells compared with WI38 cells.

The dose of the conjugator effectively tuned the killing capacity of α-Cot-NK92 cells, and monoclonal α-Cot-NK92 cell lines showed different killing capacities at different α-Cot-CAR levels. Thus, α-Cot-NK92 cells have flexible and controllable antitumor activity *via* a conjugator, without the need for re-engineering, and are readily available as a fully characterizable cell-line product without obvious risks of manufacturing failures. In conclusion, α-Cot-NK92 cells, feasible to apply Cot-conjugators linked to FDA-approved antibodies (trastuzumab), and well-established affibodies are continuously expandable off-the-shelf cell therapeutics with the flexibility to antigens. The α-Cot-NK92 cells developed in this study require pre-clinical safety tests and provide a universal CAR-NK cell platform to improve patient-specific outcomes against multi-TAA. Our data represent preliminary results that must be further evaluated in a clinical setting. α-Cot-NK92 cells might become clinically helpful for treating patient-specific tumors through multi-targeting using various conjugators to major TAAs of a patient. Furthermore, our universal CAR-NK cells are viable and a cost-effective alternative to CAR-modified patient T cells and conventional CAR-NK/T cells.

## Data availability statement

The original contributions presented in the study are included in the article/**Supplementary Material**. Further inquiries can be directed to the corresponding authors.

## Ethics statement

The animal study was reviewed and approved by Committee of the Korean Basic Science Institute(KBSI-AEC 1916) guidelines.

## Author contributions

HYK, SYL, and HMK contributed equally to this study. HYK and SYL wrote the manuscript, designed and performed the experiments. HMK, HL, MYC, HSP, S-CO, S-MK, and EHH performed the experiments. T-DK, KSH, IC, and JC designed and supervised experiments. All authors approved the final manuscript and conceptualization, supervision, review, and editing.

## References

[1] Rezvani K Rouce R Liu E Shpall E. Engineering natural killer cells for cancer immunotherapy. Mol Ther (2017) 25(8):1769–81. doi: 10.1016/j.ymthe.2017.06.012 PMC5542803 28668320

[2] Sadelain M. Chimeric antigen receptors: Driving immunology towards synthetic biology. Curr Opin Immunol (2016) 41:68–76. doi: 10.1016/j.coi.2016.06.004 27372731 PMC5520666

[3] Gajewski TF Schreiber H Fu YX. Innate and adaptive immune cells in the tumor microenvironment. Nat Immunol (2013) 14(10):1014–22. doi: 10.1038/ni.2703 PMC4118725 24048123

[4] Krneta T Gillgrass A Chew M Ashkar AA. The breast tumor microenvironment alters the phenotype and function of natural killer cells. Cell Mol Immunol (2016) 13(5):628–39. doi: 10.1038/cmi.2015.42 PMC5037278 26277898

[5] Newick K O'Brien S Moon E Albelda SM. Car T cell therapy for solid tumors. Annu Rev Med (2017) 68:139–52. doi: 10.1146/annurev-med-062315-120245 27860544

[6] Zhang BL Qin DY Mo ZM Li Y Wei W Wang YS. Hurdles of car-T cell-based cancer immunotherapy directed against solid tumors. Sci China Life Sci (2016) 59(4):340–8. doi: 10.1007/s11427-016-5027-4 26965525

[7] Ma JS Kim JY Kazane SA Choi SH Yun HY Kim MS. Versatile strategy for controlling the specificity and activity of engineered T cells. Proc Natl Acad Sci U S A (2016) 113(4):E450–8. doi: 10.1073/pnas.1524193113 PMC4743826 26759368

[8] Urbanska K Lanitis E Poussin M Lynn RC Gavin BP Kelderman S. A universal strategy for adoptive immunotherapy of cancer through use of a novel T-cell antigen receptor. Cancer Res (2012) 72(7):1844–52. doi: 10.1158/0008-5472.CAN-11-3890 PMC3319867 22315351

[9] Grote S Mittelstaet J Baden C Chan KC Seitz C Schlegel P. Adapter chimeric antigen receptor (Adcar)-engineered nk-92 cells: An off-the-Shelf cellular therapeutic for universal tumor targeting. Oncoimmunology (2020) 9(1):1825177. doi: 10.1080/2162402X.2020.1825177 33457105 PMC7781805

[10] Tamada K Geng D Sakoda Y Bansal N Srivastava R Li Z. Redirecting gene-modified T cells toward various cancer types using tagged antibodies. Clin Cancer Res (2012) 18(23):6436–45. doi: 10.1158/1078-0432.CCR-12-1449 23032741

[11] Rodgers DT Mazagova M Hampton EN Cao Y Ramadoss NS Hardy IR. Switch-mediated activation and retargeting of car-T cells for b-cell malignancies. Proc Natl Acad Sci U S A (2016) 113(4):E459–68. doi: 10.1073/pnas.1524155113 PMC4743815 26759369

[12] Yoon SR Lee YS Yang SH Ahn KH Lee JH Lee JH. Generation of donor natural killer cells from Cd34(+) progenitor cells and subsequent infusion after hla-mismatched allogeneic hematopoietic cell transplantation: A feasibility study. Bone Marrow Transplant (2010) 45(6):1038–46. doi: 10.1038/bmt.2009.304 19881555

[13] Rubnitz JE Inaba H Ribeiro RC Pounds S Rooney B Bell T. Nkaml: A pilot study to determine the safety and feasibility of haploidentical natural killer cell transplantation in childhood acute myeloid leukemia. J Clin Oncol (2010) 28(6):955–9. doi: 10.1200/JCO.2009.24.4590 PMC2834435 20085940

[14] Zahid A Siegler EL Kenderian SS. Cart cell toxicities: New insight into mechanisms and management. Clin Hematol Int (2020) 2(4):149–55. doi: 10.2991/chi.k.201108.001 PMC7785104 33409484

[15] Xue L Yi Y Xu Q Wang L Yang X Zhang Y. Chimeric antigen receptor T cells self-neutralizing Il6 storm in patients with hematologic malignancy. Cell Discovery (2021) 7(1):84. doi: 10.1038/s41421-021-00299-6 34518515 PMC8437938

[16] Murthy H Iqbal M Chavez JC Kharfan-Dabaja MA. Cytokine release syndrome: Current perspectives. Immunotargets Ther (2019) 8:43–52. doi: 10.2147/ITT.S202015 31754614 PMC6825470

[17] Siegler EL Kenderian SS. Neurotoxicity and cytokine release syndrome after chimeric antigen receptor T cell therapy: Insights into mechanisms and novel therapies. Front Immunol (2020) 11:1973. doi: 10.3389/fimmu.2020.01973 32983132 PMC7485001

[18] Kim H Yoon S Chung J. *In vitro* and in vivo application of anti-cotinine antibody and cotinine-conjugated compounds. BMB Rep (2014) 47(3):130–4. doi: 10.5483/bmbrep.2014.47.3.006 PMC4163880 24499668

[19] Cho JH Collins JJ Wong WW. Universal chimeric antigen receptors for multiplexed and logical control of T cell responses. Cell (2018) 173(6):1426–38.e11. doi: 10.1016/j.cell.2018.03.038 29706540 PMC5984158

[20] Raj D Yang MH Rodgers D Hampton EN Begum J Mustafa A. Switchable car-T cells mediate remission in metastatic pancreatic ductal adenocarcinoma. Gut (2019) 68(6):1052–64. doi: 10.1136/gutjnl-2018-316595 PMC6580747 30121627

[21] Mitwasi N Feldmann A Arndt C Koristka S Berndt N Jureczek J. "Unicar"-modified off-the-Shelf nk-92 cells for targeting of Gd2-expressing tumour cells. Sci Rep (2020) 10(1):2141. doi: 10.1038/s41598-020-59082-4 32034289 PMC7005792

[22] Kim N Kim M Yun S Doh J Greenberg PD Kim TD. Microrna-150 regulates the cytotoxicity of natural killers by targeting perforin-1. J Allergy Clin Immunol (2014) 134(1):195–203. doi: 10.1016/j.jaci.2014.02.018 24698324 PMC4125537

[23] Kalos M Levine BL Porter DL Katz S Grupp SA Bagg A. T Cells with chimeric antigen receptors have potent antitumor effects and can establish memory in patients with advanced leukemia. Sci Transl Med (2011) 3(95):95ra73. doi: 10.1126/scitranslmed.3002842 PMC3393096 21832238

[24] Davila ML Riviere I Wang X Bartido S Park J Curran K. Efficacy and toxicity management of 19-28z car T cell therapy in b cell acute lymphoblastic leukemia. Sci Transl Med (2014) 6(224):224ra25. doi: 10.1126/scitranslmed.3008226 PMC4684949 24553386

[25] Hsu JL Hung MC. The role of Her2, egfr, and other receptor tyrosine kinases in breast cancer. Cancer Metastasis Rev (2016) 35(4):575–88. doi: 10.1007/s10555-016-9649-6 PMC5215954 27913999

[26] Hirsch FR Varella-Garcia M Cappuzzo F. Predictive value of egfr and Her2 overexpression in advanced non-Small-Cell lung cancer. Oncogene (2009) 28 Suppl 1:S32–7. doi: 10.1038/onc.2009.199 19680294

[27] Klingemann HG. Cellular therapy of cancer with natural killer cells-where do we stand? Cytotherapy (2013) 15(10):1185–94. doi: 10.1016/j.jcyt.2013.03.011 23768925

[28] Geller MA Miller JS. Use of allogeneic nk cells for cancer immunotherapy. Immunotherapy (2011) 3(12):1445–59. doi: 10.2217/imt.11.131 PMC3292871 22091681

[29] Oberoi P Wels WS. Arming nk cells with enhanced antitumor activity: Cars and beyond. Oncoimmunology (2013) 2(8):e25220. doi: 10.4161/onci.25220 24167761 PMC3805653

[30] Klingemann H. Are natural killer cells superior car drivers? Oncoimmunology (2014) 3:e28147. doi: 10.4161/onci.28147 25340009 PMC4203506

