# Supplementary material for: A modifiable universal cotinine-chimeric antigen system of NK cells with multiple targets
Source: Front Immunol. 2023 Jan 13;13:1089369. doi: 10.3389/fimmu.2022.1089369 (PMC9880444; doi:10.3389/fimmu.2022.1089369)
Supplement: Supplementary file 1 [file DataSheet_1.pdf]

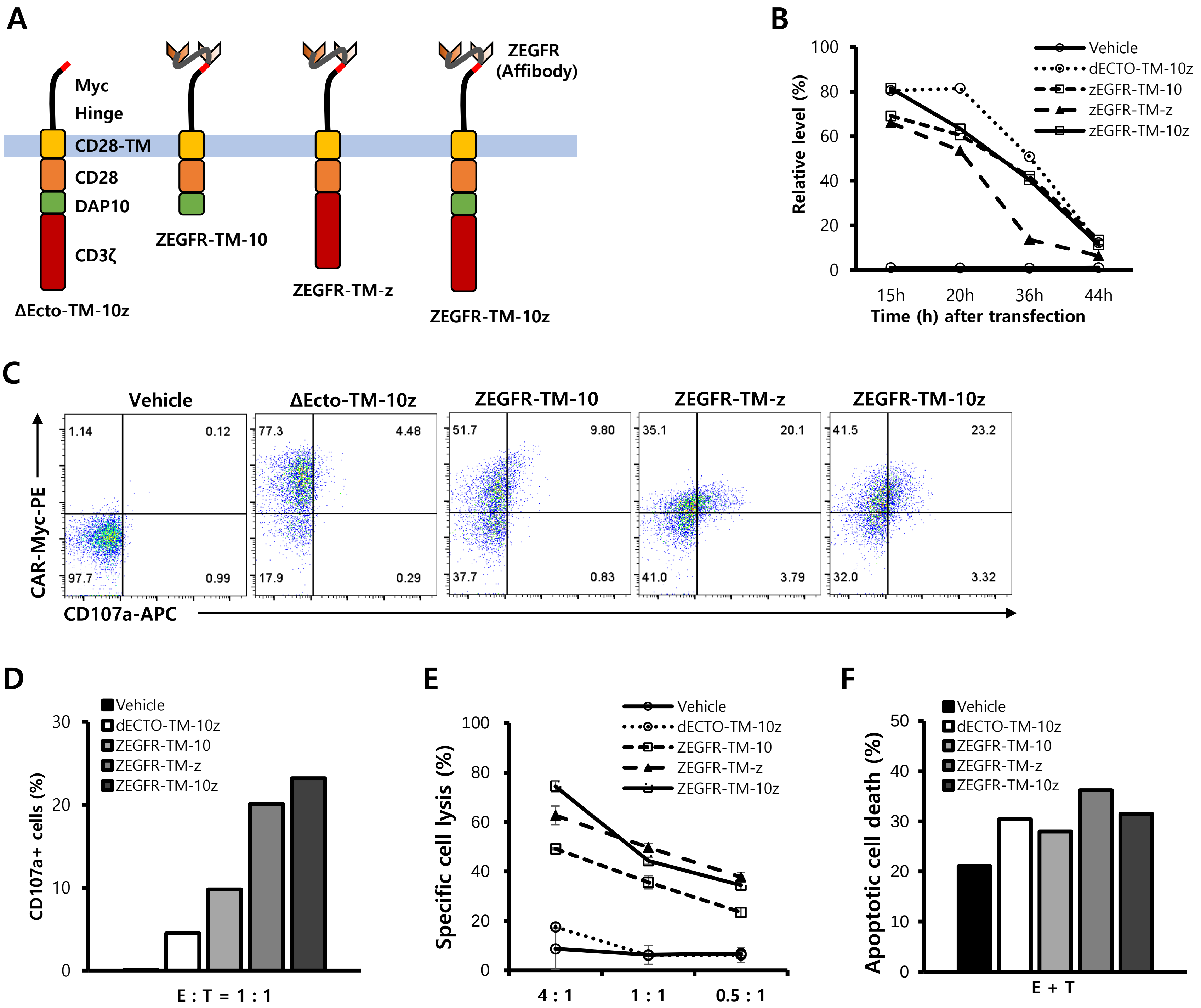

Supplementary Figure 1. Function of each domain in CAR structure through transient expression of ZEGFR-CAR using mRNA transfection method. (A) The structure of ZEGFR-CAR;Δecto-TM-10z, ZEGFR-TM-10, ZEGFR-TM-z, ZEGFR-TM-10z. **(B)** Time-dependent expression levels of five CAR types after CAR mRNA transfection. **(C and D)** Expression of CD107a in vehicle and five types of transient CAR-NK cells through FACS analysis. Transient CAR-NK cells were co-cultured with AU565 cells at a 1:1 E:T ratio for 4 h at 15 h after transfection and then were labeled with CD56-BV, Myc-PE, and CD107-APC antibodies. **(E)** Cytotoxicity of transient CAR-NK cells at various E:T ratios (4:1, 1:1, and 0.5:1) at 15 h after transfection. **(F)** Apoptotic cell death in transient CAR-NK cells. Transient expression of CAR increased apoptosis compared with vehicle group.

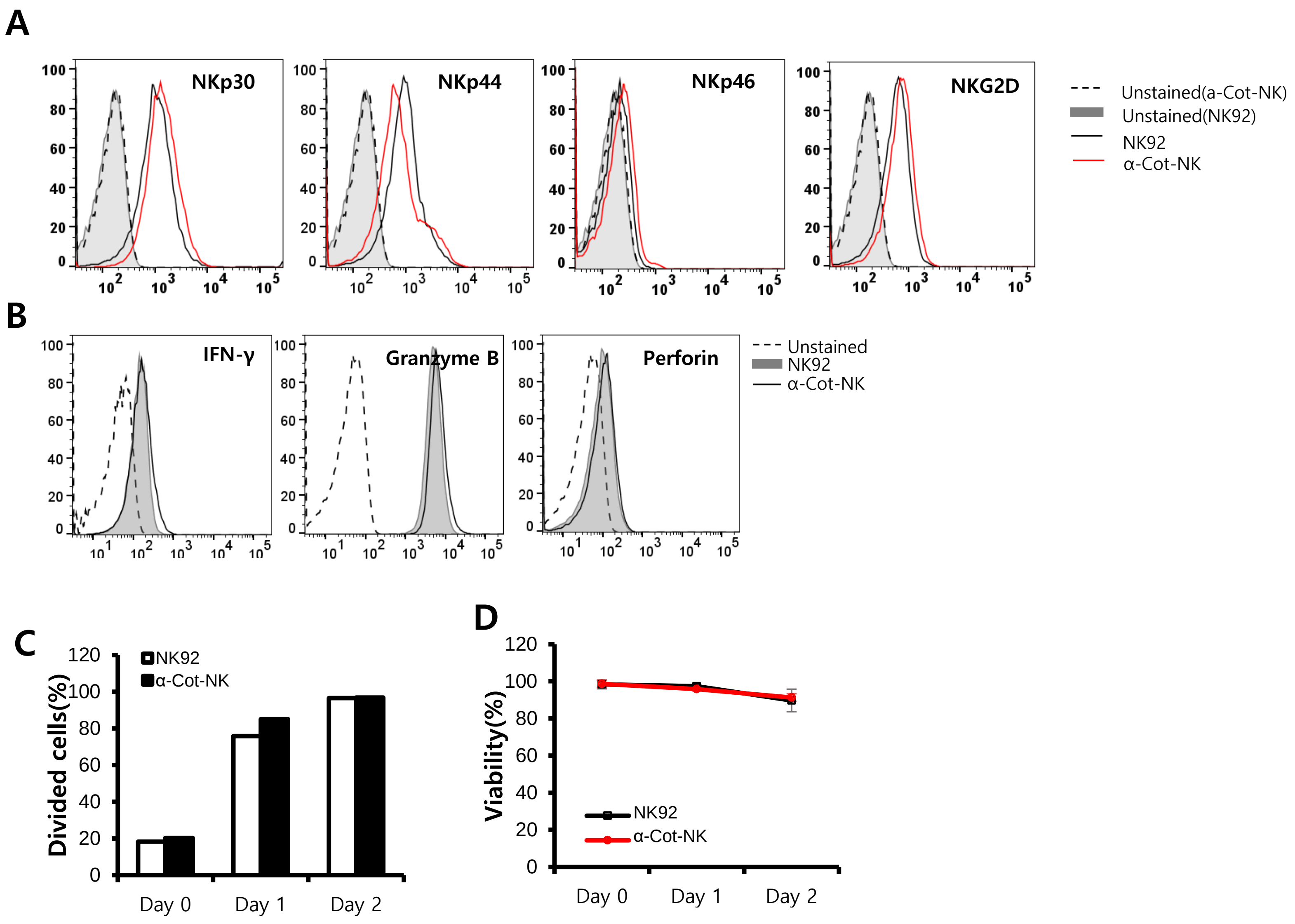

Supplementary Figure 2. Generation of α-Cot-NK cells. (A and B) Expression profiling of activation receptors **(A)** and functional markers **(B)** in parental NK92 and α-Cot-NK cells. Expression levels of activation receptors (NKp30, NKp44, NKp46, and NKG2D) and functional markers (IFN-γ, granzyme B, and perforin) in α-Cot-NK cells were identified using FACS and compared with those in parental NK92 cells. **(C and D)** Proliferative ability **(C)** and viability **(D)** of α-Cot-NK cells compared with those of parental NK92 cells. Proliferation ability indicates the percentage of dividing cells relative to α-Cot-NK cells stained with violet tracer confirmed using FACS for 2d. Viability of parental NK92 and α-Cot-NK cells was measured through trypan blue staining for 2d. These values are representative of at least three independent experiments. Cot, cotinine; NK, natural killer.

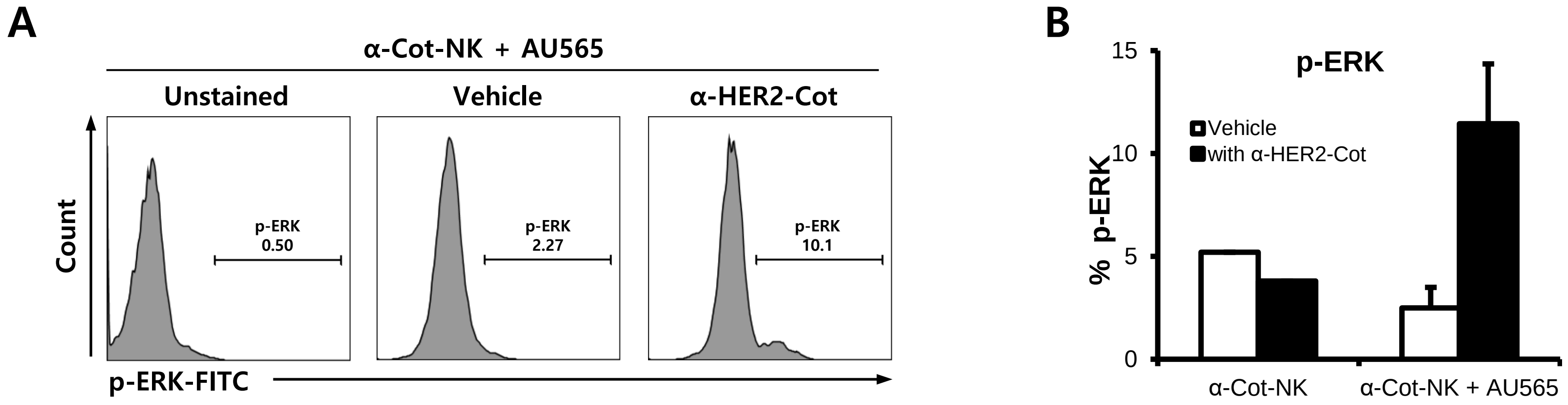

Supplementary Figure 3. Activation of ERK signaling by stimulation with AU565 cells with/without conjugators. (A and B) α-Cot-NK cells were co-cultured with/without AU565 cells and with/without conjugators and were then intracellularly stained for p-ERK. Data measured using flow cytometry are presented as a histogram **(A)** and graph **(B)** as the percentage of p-ERK. Cot, cotinine; NK, natural killer.

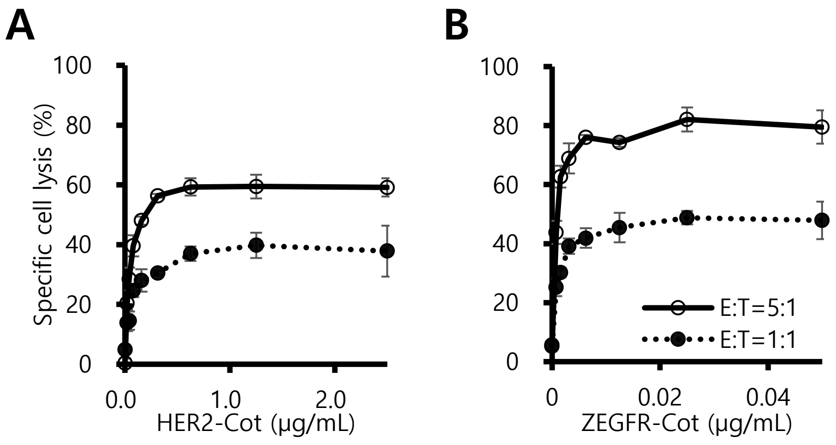

Supplementary Figure 4. Cytotoxicity according to the amount of conjugator. (A and B) Effect of parameters, such as type and dose of conjugator, on cytotoxicity. Ability of α-Cot-NK cells to lyse AU565 cells was assessed using co-culture for 4 h at E:T ratios of 5:1 and 1:1 in a dose-dependent manner of antibody (α-HER2) **(A)** or affibody (ZEGFR) **(B)** conjugated with Cot. α-HER2-Cot or ZEGFR-Cot was diluted in PBS at a 1/2 dilution factor from the highest concentration of 2.5 μg/mL to 0.0195 μg/mL or from the highest concentration of 0.05 μg/mL to 0.00078 μg/mL, respectively.

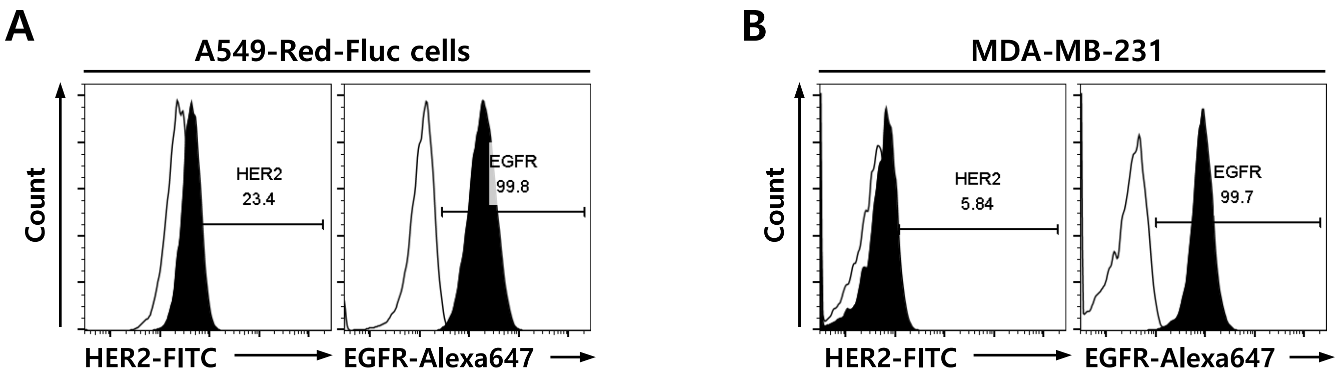

Supplementary Figure 5. Ligand levels in tumor cell lines. (A and B) Expression levels of HER2 and EGFR in A549-Red-Fluc (A) and MDA-MB-231 (B) cells were identified using FACS.

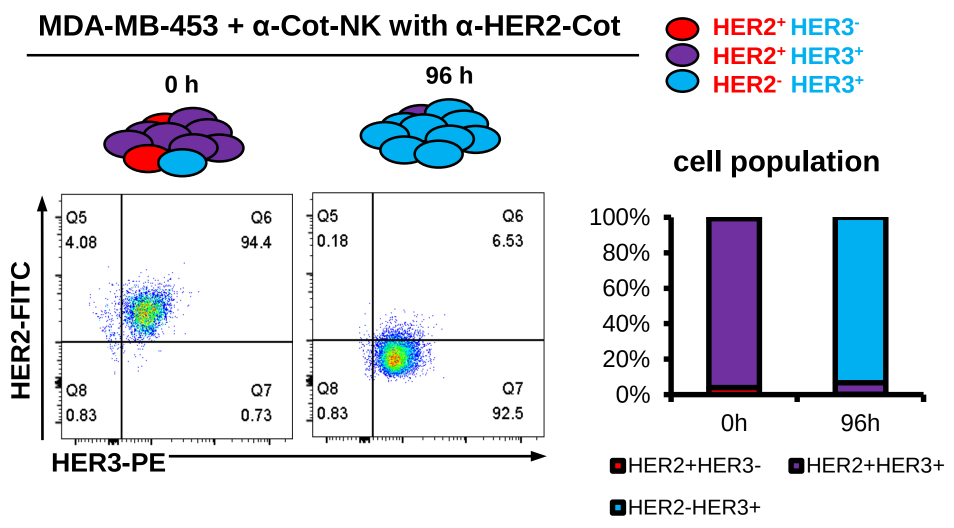

Supplementary Figure 6. Changes of population in tumor cell line. A plot indicating the change in the population (left) and percentage (right) of HER2+HER3+ MDA-MB-453 cells evaluated at 4d, after treatment with α-Cot-NK92 cells with HER2-Cot for 4 h

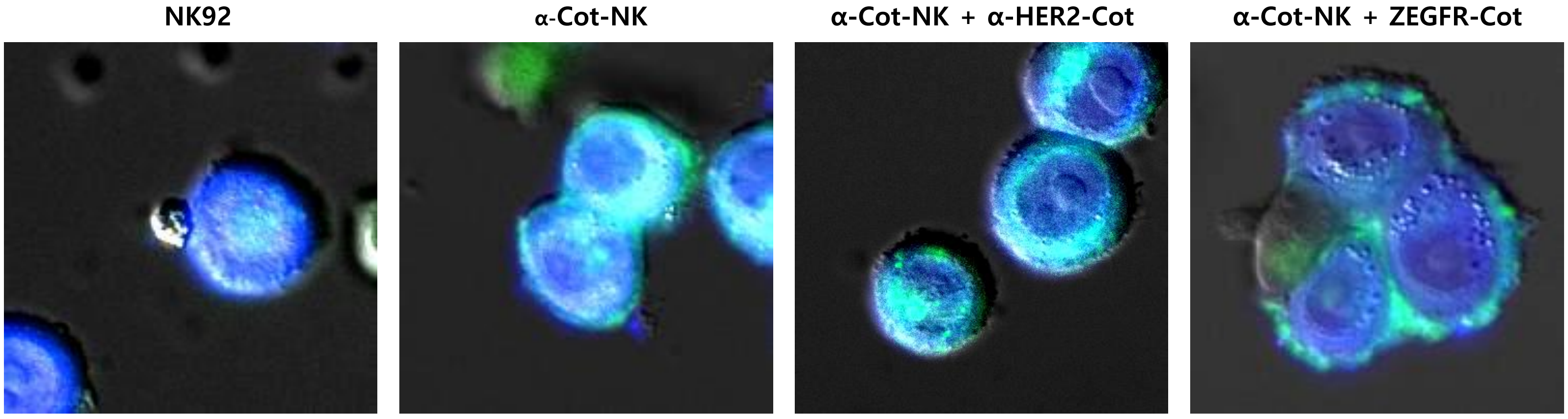

Lysosensor: NK

Propidium Iodide : Dead cell

Cell trace far red: AU565

Supplementary Movie 1. Cytolytic action on immune synapse when α-Cot-NK cell comes into contact with AU565 cell.

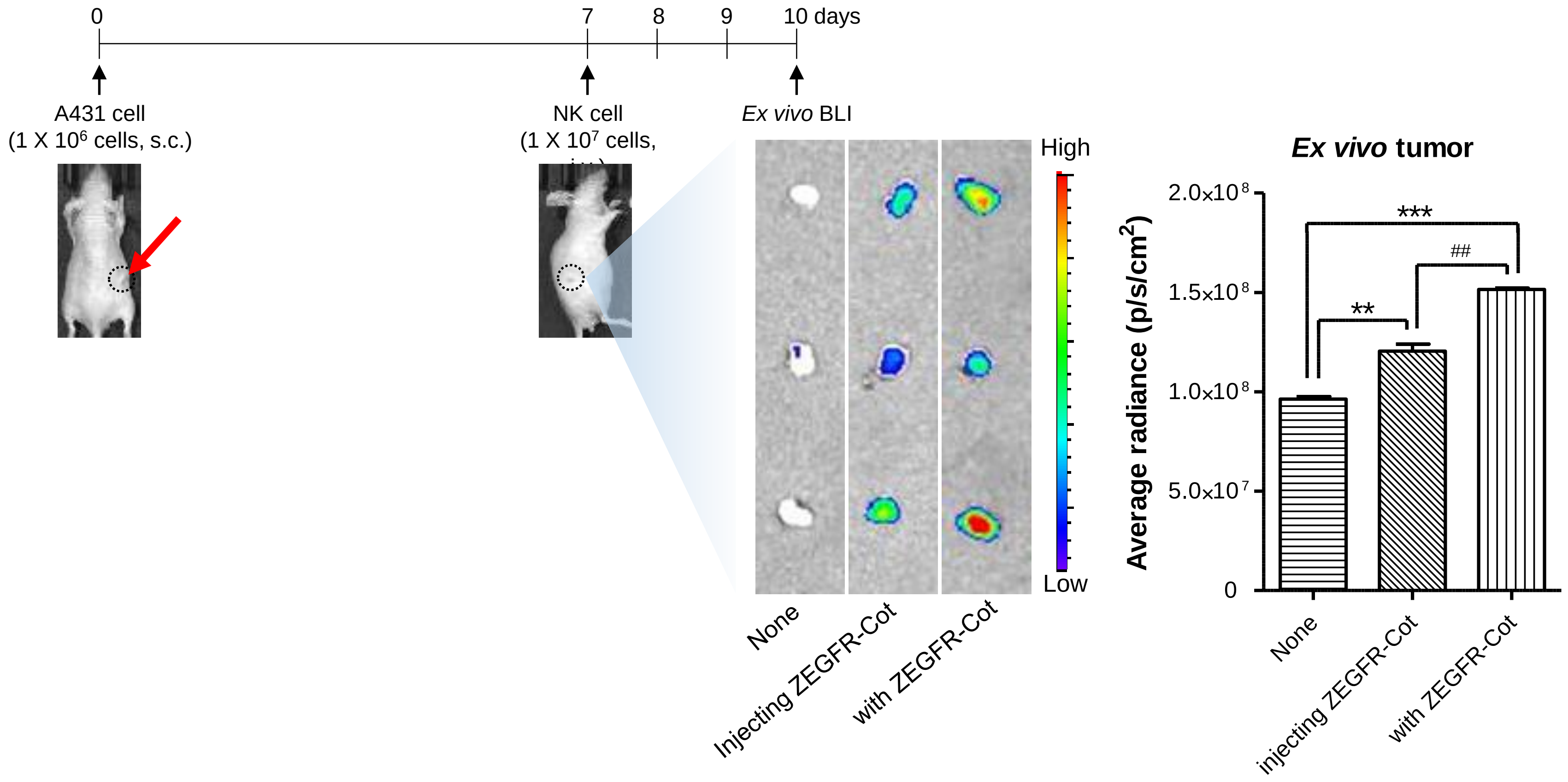

Supplementary Figure 7. Tumor targeting of DiR-staining NK92/α-Cot-NK cells. A431 cells were subcutaneously inoculated into the flanks of nude mice. Seven days after tumor inoculation, DiR-labeled NK92 (None) or α-Cot-NK cells with/without ZEGFR-Cot were intravenously injected through the tail vein. To assess the tumor-targeting and NK-binding potential by a conjugator in the *in vivo* model, one group administrated ZEGFR-Cot at 22 h after injection of α-Cot-NK cells without ZEGFR-Cot. Tumors were extracted from the right flank, and photon intensity was monitored at 72 h after NK cell injection. Average radiance data were plotted as the mean ± S.D (n = 3). Cot, cotinine; NK, natural killer.
